# Reduced Risk for Metabolic Syndrome and Insulin Resistance Associated with Ovo-Lacto-Vegetarian Behavior in Female Buddhists: A Case-Control Study

**DOI:** 10.1371/journal.pone.0071799

**Published:** 2013-08-09

**Authors:** Jui-Kun Chiang, Ying-Lung Lin, Chi-Ling Chen, Chung-Mei Ouyang, Ying-Tai Wu, Yu-Chiao Chi, Kuo-Chin Huang, Wei-Shiung Yang

**Affiliations:** 1 Department of Family Medicine, Dalin Tzuchi Hospital, Buddhist Tzuchi Medical Foundation, Chiayi, Taiwan; 2 Graduate Institute of Clinical Medicine, College of Medicine, National Taiwan University, Taipei, Taiwan; 3 Graduate Institute of Physical Therapy, College of Medicine, National Taiwan University, Taipei, Taiwan; 4 Graduate Institute of Epidemiology, College of Public Health, National Taiwan University, Taipei, Taiwan; 5 Department of Dietetics, National Taiwan University Hospital, Taipei, Taiwan; 6 Department of Family Medicine, National Taiwan University Hospital, Taipei, Taiwan; 7 Department of Internal Medicine, National Taiwan University Hospital, Taipei, Taiwan; University College London, United Kingdom

## Abstract

**Introduction:**

The association of vegetarian status with the risk of metabolic syndrome (MetS) is not clear. In Asia, Buddhists often have vegetarian behavior for religious rather than for health reasons. We hypothesize that the vegetarian in Buddhism is associated with better metabolic profiles, lower risk for the MetS and insulin resistance (IR).

**Methods:**

We enrolled 391 female vegetarians (∼80% lacto-ovo-vegetarians) and 315 non-vegetarians from health-checkup clinics at a Buddhist hospital in Taiwan.

**Results:**

The vegetarian status was associated with lower body mass index, smaller waist circumference, lower total cholesterol, lower low density lipoprotein-cholesterol (LDL-C), and lower HDL-C in multivariate linear regression analyses. Despite having lower HDL-C level, the vegetarians had significantly lower total cholesterol/HDL-C and LDL-C/HDL-C ratios. After adjusting the other covariates, the risks for the MetS were lower for ovo-lacto-vegetarians of 1–11 years and >11 years respectively by 54% (odds ratio [OR] = 0.46, 95%C.I.:0.26–0.79) and 57% (OR = 0.43, 95%C.I.:0.23–0.76) compared to non-vegetarians by the IDF criteria. Likewise, they were lower respectively by 45% (OR = 0.55, 95%C.I.:0.32–0.92) and 42% (OR = 0.58, 95%C.I.:0.33–0.997), for the MetS by the modified NCEP criteria. In the subgroup of non-diabetic subjects, the vegetarians also had lower risk for IR by HOMA compared to the non-vegetarians (OR = 0.71, 95%C.I.:0.48–1.06).

**Conclusion:**

The vegetarian behavior, mainly lacto-ovo-vegetarian, related to Buddhism, although not meant for its health effects, is associated with reduced risk for the MetS and IR and may potentially provide metabolic and cardiovascular protective effects in women.

## Background

Obesity has become a serious global health issue especially in the developed countries. Many chronic disorders such as metabolic syndrome (MetS), diabetes mellitus (DM), hypertension, and coronary artery disease are closely associated with obesity. Lifestyle modification has been shown to be effective in reducing obesity and its comorbidity such as type 2 DM [1]–[5]. Vegetarian diet is one way of lifestyle modifications that may render beneficial metabolic effects [6].

Vegetarians often have better metabolic profiles compared to non-vegetarians in previous studies. Vegetarians were reported to have lower body weight [7], lower body mass index (BMI) [8], lower plasma glucose [9], lower blood pressures [10], and lower levels of total cholesterol [7] and low density lipoprotein-cholesterol (LDL-C) [11]. Yet, vegetarians were also documented to have lower levels of high density lipoprotein-cholesterol (HDL-C) and higher levels of triglycerides [9]. Thus, it is not clear whether vegetarian status is associated with reduced risk of the MetS, until recently Rizzo *et al*. reported an association of vegetarian dietary patterns with reduced risk of the MetS [12].

In Asia, many people especially females adopt vegetarian diet for religious belief, such as Buddhism. Animal products except milk and egg are strictly prohibited in diet. Whether this kind of dietary behavior, based on religious rather than health reasons, is associated with reduced risk for the MetS is not clear. In this study, we examined the association of vegetarian, mainly lacto-ovo-vegetarian, status with the MetS and insulin resistance (IR) in Taiwanese female vegetarians associated with Buddhism. We also analyzed the effect of vegetarian duration on these risks.

## Methods

### Subjects

A total of 706 female subjects, including 391 vegetarians and 315 age-matched non-vegetarians were included in this study. The participants were volunteer helpers of Buddhist Tzu Chi Foundation from southern Taiwan, including Yulin, Chiayi, Tainan, and Kaoshiung counties close to the hospital where the study was conducted. Many of this volunteer program are vegetarians. They were recruited when they took the general health examination as a part of their benefit of this volunteer program at the Dalin Tzuchi Hospital, the Buddhist Tzuchi Medical Foundation, at Chia-Yi county between May 2007 and April 2008. Subjects with severe systemic diseases, such as cancer, heart failure, uremia and liver cirrhosis or acute illness, such as acute myocardial infarction were not recruited. Among these 760 females, 36, 138, and 3 had previous diagnosis of diabetes, hypertension and hyperlipidemia respectively. Male vegetarians were also recruited, but the number was much less, therefore were not included for analysis. An age frequency-matched process was performed to select the subjects from the non-VEG group (*n*  =  402) for balancing the age distribution between the VEG and non-VEG groups. Specifically, we stratified the subjects in both VEG and non-VEG groups into four 10-year range age groups (< 50, 50-<60, 60-<70, and 70 and +). In each age group, we randomly selected the subjects in the non-VEG group in such a way that the ratio between the subjects of the VEG and non-VEG groups was 0.8. This study was approved by the Institutional Review Board of the Dalin Tzuchi Hospital (Nos. B09504005, B09604003-1). Written informed consent was obtained from each subject.

Vegetarian status was determined by investigators based on self-reported questionnaire. The questionnaire was filled by the subjects themselves with the help from our research assistants. Dietary questionnaire included vegetarian status, egg intake, milk intake, vegetarian duration, times of vegetarian diet in a day and how many days of vegetarian diet in a month. Many Buddhists consider themselves as “vegetarian”, even they practice only one vegetarian meal everyday or only on special days of the month based on lunar calendar. These were classified as non-vegetarian in this study. Subjects who practiced vegetarian diet at all meals daily and persistently for more than 1 year were defined as vegetarians; otherwise, they were considered as non-vegetarians. Those who did not consume any animal product at all were “pure vegetarians,” whereas those who consumed egg and/or milk for animal protein supplement were respectively “ovo-”, “lacto-”, and “ovo-lacto-vegetarians”. Among the 391 vegetarians, there were 22 pure vegetarians (5.6%), 20 ovo-vegetarians (5.1%), 33 lacto-vegetarians (8.4%), and 316 ovo-lacto-vegetarians (80.9%). There were no smoker and only 12 social alcohol drinkers in this study, probably because they were associated with a specific religious group. Daily activity was assessed by a 7-day recalled physical activity questionnaire (Chinese version) as previously described [13]–[15]. Briefly, the activity of weekdays and weekend were treated separately. Mean sleep hours, work hours on work days, work contents, exercise hours, exercise contents, usual activity hours and contents were recorded. The ratio of work metabolic rate to resting metabolic rate of each activity can be obtained. Finally the energy expenditure in kilocalories or kilocalories per kilogram body weight can be estimated for all activities, special activities, or activity types. Anthropometric data, including weight, height, and waist circumference were recorded. Body weight and height were measured while subjects were minimally clothed without shoes using digital scales (BW-110, Yuang Tong Weight Scale Factory Co., LTD., New Taipei City, Taiwan). Waist circumference was measured at the umbilical level using a tape.

Sitting blood pressure (BP) was measured by an automatic blood pressure machine (ProCare 100, GE Medical System Information Technologies, Inc., Piscataway, NJ, U.S.A.). If the subjects had BP over 130/85 mmHg, BP was measured again after 15–30 minute rest. The second BP was used for analysis. According to the cut-off values recommended by WHO for Asians, central obesity was defined as the waist circumference ≥80 cm and obesity was defined by BMI ≥25 kg/m^2^ in this study.

### Laboratory tests

The blood sample was collected after 8-hour fasting for laboratory tests of plasma glucose, serum insulin and serum lipids. Fasting plasma glucose, and serum lipids including total cholesterol, HDL-C, LDL-C, and triglycerides were assayed with spectrophotometric method (Roche Integra 800, Roche Modular PP, Clinical Diagnostic Inc., Johonson and Johonson Company, Germany) in hospital central lab. Hypercholesterolemia was defined as the level of total cholesterol ≥6.22 mmol/L (240 mg/dL). Hypertriglyceridemia was defined as the level of triglyceride ≥1.70 mmol/L (150 mg/dL). Hyper LDL-C was defined as the level of LDL-C ≥4.14 mmol/L (160 mg/dL). And, hypo HDL-C was defined as the level of HDL-C <1.30 mmol/L (50 mg/dL). The cut-off values were chosen based on the cut-off values recommended in National Cholesterol Education Program (NCEP) ATP-III [16]. The modified NCEP definition of MetS for Asians is composed of three out of the following five items: (1) fasting glucose level >5.55 mmol/L (100 mg/dL) or known diabetes with anti-diabetic treatment, (2) blood pressure >130/85 mmHg or known hypertension with anti-hypertensive treatment, (3) fasting serum triglyceride level ≥1.70 mmol/L (150 mg/dL), (4) fasting HDL cholesterol level <1.30 mmol/L (50 mg/dL), and (5) waist circumference >80 cm [17]. The International Diabetes Federation (IDF) definition of the MetS includes the central obesity by waist circumference plus two of the other criteria listed above. Blood samples were stored in –80°C before insulin assay. Human insulin ELISA kit (BioSource Europe S.A., Nivelles, Belgium) was used for insulin assay. The HOMA model was used for IR evaluation [18], but the cases with known diabetes were excluded when using this index. IR was arbitrarily defined as the value of HOMA-IR larger than the third quartile of its empirical distribution — that is, HOMA-IR ≥2 in this study, which was consistent with some of the other studies [19]. The following definitions and diagnostic criteria for diabetes, impaired fasting glucose (IFG), and hypertension were listed as below: Diabetes: receiving medications for diabetes or fasting glucose levels ≥ 126 mg/dL; IFG: fasting glucose between 100 mg/dL and 126 mg/dL; hypertension: receiving medications for hypertension, systolic blood pressure > 140 mmHg, or diastolic blood pressure > 90 mmHg.

### Statistical Analysis

Data analysis was performed using the SAS 9.1.3 (SAS Institute Inc., Cary, NC, U.S.A.) and R 2.9.2 (Free Software Foundation, Inc., Boston, MA, U.S.A.) software. In statistical testing, two-sided *p* value ≤ 0.05 was considered statistically significant. Continuous data were expressed as mean ± standard deviation (SD) unless otherwise indicated. Categorical variables were represented by frequency and percentage (%). Normality of data distribution was checked with the Shapiro-Wilk test. Log transformation was performed for the variables with skewed distributions such as TG and HOMA before statistical testing. Two-sample *t* test was applied to examine the differences in means of continuous variables between the vegetarian and non-vegetarian groups and χ^2^ test was used to assess the association of each categorical variable with the vegetarian vs. non-vegetarian groups. Multivariate linear regression analysis and logistic regression analysis were conducted to examine the associations between vegetarian status and various anthropometric and metabolic factors including BMI, waist circumference, glucose, systolic blood pressure, triglycerides, LDL-C, HDL-C, and HOMA-IR respectively after adjusting for known confounding variables.

Basic model-fitting techniques for regression analysis, including (1) variable selection, (2) goodness-of-fit (GOF) assessment, and (3) regression diagnostics (e.g., residual analysis, detection of influential cases, and check for multicollinearity), were applied to assure the quality of analysis results. The variables on the list to be selected during the variable selection procedure were listed in Table 1. Two approaches to variable selection were applied: (1) subjective variable selection procedure based on pathophysiologic knowledge and (2) stepwise variable selection procedure with iterations between the forward and backward steps. In stepwise variable selection procedure, both significance level for entry and significance level for stay were set to 0.15 for conservativeness. The GOF measures including the coefficient of determination *R* ^2^ (for linear regression model), the area under receiver operating characteristic curve (AUC), and the adjusted generalized *R* ^2^ (for logistic regression model) and the Hosmer-Lemeshow GOF test (for logistic regression model) were examined. The coefficient of determination (*R* ^2^) is the square of the Pearson correlation between the observed and predicted values of the continuous response variable in linear regression model. A larger value of *R* ^2^ indicates a better fit of linear regression model. Larger *p* values of the Hosmer-Lemeshow GOF test indicate better fits of logistic regression model. According to Hosmer and Lemshow (2000), as a general rule, AUC of 0.7 and above is considered acceptable discrimination. And, the statistical tools for regression diagnostics such as residual analysis, detection of influential cases, and check for multicollinearity were applied to discover model or data problems.

**Table 1:** Comparison of the basic characteristics between the vegetarian and non-vegetarian groups.

		Vegetarians	Non-Vegetarians	*P* value
Variables	Total	(*n* = 391)	(*n* = 315)	
Age (years)	56.4±8.4	56.4±8.9	56.4±7.6	0.880
Menopause, *n* (%)	495 (70.1%)	272 (69.6%)	223 (70.8%)	0.723
BMI (kg/m^2^)	23.3±3.0	22.9±2.7	23.8±3.2	<0.001
BMI ≥ 25 kg/m^2^, *n* (%)	180 (25.5%)	84 (21.5%)	96 (30.5%)	0.007
Waist (cm)	74.0±7.3	72.9±6.9	75.3±7.6	<0.001
Waist ≥ 80 cm, *n* (%)	137 (19.4%)	55 (14.1%)	82 (26.0%)	0.001
Glucose (mmol/L)	5.06±0.93	4.98±0.89	5.15±0.97	<0.001
Insulin (pmol/L)	46.53±39.59	41.67±37.50	52.09±41.67	<0.001
HOMA-IR*^+^	1.5±1.3	1.3±1.2	1.7±1.5	<0.001
IFG ± DM, *n* (%)	103 (14.6%)	47 (12.0%)	56 (17.8%)	0.031
Diabetes, *n* (%)	42 (6.0%)	19 (4.9%)	23 (7.3%)	0.173
Systolic BP (mmHg)	125.0±19.5	123.5±18.9	127.8±20.2	0.048
Diastolic BP (mmHg)	71.7±11.3	71.4±11.0	72.2±11.8	0.313
Hypertension, *n* (%)	240 (34.0%)	125 (32.0%)	115 (36.5%)	0.206
Total cholesterol (mmol/L)	4.91±0.90	4.68±0.83	5.21±0.90	<0.001
HDL-C (mmol/L)	1.48±0.38	1.43±0.35	1.53±0.39	0.001
LDL-C (mmol/L)	3.20±0.80	3.01±0.74	3.43±0.81	<0.001
Triglyceride (mmol/L^+^)	1.21±0.73	1.22±0.77	1.20±0.68	0.591
Hyper T-Chol (≥ 240), *n* (%)	51 (7.2%)	15 (3.8%)	36 (11.4%)	<0.001
Hypo HDL-C (< 50), *n* (%)	228 (32.3%)	140 (35.8%)	88 (27.9%)	0.026
Hyper LDL-C (≥ 160), *n* (%)	76 (10.8%)	20 (5.1%)	56 (17.8%)	<0.001
Hyper TG (≥ 150), *n* (%)	121 (17.1%)	59 (15.1%)	62 (19.7%)	0.107
T-Chol/HDL-C	3.5±0.9	3.4±0.9	3.6±0.9	0.030
LDL-C/HDL-C	2.3±0.8	2.2±0.7	2.4±0.8	0.012
TG/HDL-C^+^	2.1±1.8	2.2±2.0	2.0±1.5	0.242
MNCEP MetS, *n* (%)	115 (16.3%)	52 (13.3%)	63 (20.0%)	0.017
IDF MetS, *n* (%)	66 (9.4%)	26 (6.7%)	40 (12.7%)	0.006
Activity (× 10^3^ Kcal/day)	2.7±1.0	2.7±0.9	2.8±1.1	0.745

BMI  =  body mass index, BP  =  blood pressure, HDL-C  =  high density lipoprotein-cholesterol, LDL-C  =  low density lipoprotein-cholesterol, T-Chol  =  total cholesterol, TG  =  triglyceride, HOMA-IR  =  homeostasis model assessment-insulin resistance index, MNCEP MetS  =  modified national cholesterol education program criteria of the metabolic syndrome, IDF MetS  =  International Diabetes Federation criteria of the metabolic syndrome, and IFG ± DM  =  impaired fasting glucose ± diabetes mellitus, * diabetics were excluded. + The original numbers were shown despite log transformation before statistical tests.

## Results

The basic characteristics of 706 female subjects were shown in Table 1. Among the vegetarians, 158 subjects practiced 2–6 years, 79 subjects 7–11 years, 81 subjects 12–16 years, and 73 more than 16 years. The mean age of the vegetarian (VEG) group was not significantly different from that of the non-vegetarian (non-VEG) group, neither was the menopause status. The VEG group had significantly lower body mass index (BMI), waist circumference, systolic blood pressure (but not diastolic blood pressures), fasting plasma glucose, serum insulin level, and HOMA-IR (Table 1). The VEG group also had significantly lower prevalence rates of obesity, central obesity, and impaired fasting glucose than the non-VEG group (Table 1). There were 36 (5.10%) previously known diabetes, 6 (0.85%) newly-diagnosed diabetes cases, 49 (6.94%) newly-diagnosed IFG cases, 138 (19.55%) previously known hypertension and 102 (14.45%) newly-diagnosed hypertension cases. There was no significant difference in the prevalence of diabetes and hypertension between these two groups (Table 1).

With regard to lipid profiles, the VEG group had significantly lower levels in total cholesterol (T-Chol), LDL-C, and HDL-C than the non-VEG group (Table 1). Thus, the VEG group had less numbers in hypercholesterolemia and hyper-LDL-C but more with hypo-HDL-C (Table 1). In contrast, the triglyceride level was not significantly different between the VEG and non-VEG groups. Thus, the prevalence of hypertriglyceridemia was approximately the same between them. In terms of lipid ratios, the VEG group had lower T-Chol/HDL-C and LDL-C/HDL-C ratios than the non-VEG groups. Yet, there was no significant difference in TG/HDL-C ratio between them (Table 1).

The VEG group also had better insulin sensitivity indicated by lower HOMA-IR (Table 1). This result remained the same even after including the subjects with known diabetes in the analysis (data not shown). The prevalence of the MetS according to either the modified NCEP definition or the IDF definition was also significantly lower in the VEG group (Table 1). Taken together, the VEG group in general had better metabolic profiles than the non-VEG group.

Next, whether being vegetarian was still associated with better metabolic profiles, after adjusting for age, menopause, physical activity, and the other anthropometric and metabolic variables, was further examined respectively in linear (Table 2) and logistic regression analyses (Tables 3) based on pathophysiologic knowledge. The VEG was significantly associated with lower BMI (Table 2) and reduced risk for obesity (Table 3) compared to the non-VEG group, after adjusting for age, fasting plasma glucose, triglyceride, menopause, and activity score. The VEG was significantly associated with smaller waist circumference (Table 2) and reduced risk for central obesity (Table 3), compared to the non-VEG group after adjusting for age, fasting plasma glucose, triglyceride, menopause, and activity score. Similarly, the VEG group had a significantly lower HOMA-IR (Table 2) and reduced risk for IR of borderline significance (Table 3) compared to the non-VEG group after adjusting for age, menopause, waist, glucose, and triglyceride and activity with the exclusion of diabetic cases.

**Table 2:** Multiple linear regression analyses of the associations between vegetarian status and selected metabolic parameters as outcome variables using the subjective variable selection procedure based on pathophysiologic knowledge.

				Outcome	variable			
	BMI	Waist	Glucose	SBP	log(TG)	LDL-C	HDL-C	logHOMA-IR*
Covariate								
Vegetarian (yes vs. no)	–0.92±0.21	–2.40±0.50	–0.13±0.06	–1.77±1.37	0.06±0.03	–0.41±0.06	–0.10±0.02	–0.25±0.06
	(< 0.001)	(< 0.001)	(0.025)	(0.197)	(0.061)	(< 0.001)	(< 0.001)	(<0.001)
Age (years)	0.01±0.02	0.12±0.04	0.01±.005	0.67±0.11	0.01±.003	–.003±.005	.002±.002	–0.01±0.01
	(0.415)	(0.002)	(0.168)	(< 0.001)	(0.011)	(0.504)	(0.282)	(0.007)
Menopause (yes vs. no)	–0.12±0.29	0.30±0.71	–0.01±0.08	–2.77±1.90	–0.05±0.05	0.15±0.08	0.04±0.03	–0.10±0.09
	(0.681)	(0.673)	(0.914)	(0.145)	(0.342)	(0.068)	(0.233)	(0.266)
Waist (cm)	–	–	0.01±.004	0.25±0.10	0.02±.003	0.01±.004	–0.01±.002	0.03±0.05
			(0.053)	(0.017)	(< 0.001)	(0.119)	(< 0.001)	(< 0.001)
Glucose (mmol/L)	0.15±0.12	0.62±0.29	–	0.91±0.78	0.12±0.02	0.02±0.03	–.005±0.01	0.32±0.06
	(0.201)	(0.032)		(0.241)	(< 0.001)	(0.641)	(0.730)	(< 0.001)
log(Triglyceride)	1.55±0.23	3.60±0.55	0.31±0.07	–1.17±1.54	–	0.40±0.07	–0.34±0.03	0.30±0.07
(log mmo/L)	(< 0.001)	(< 0.001)	(< 0.001)	(0.447)		(<0.001)	(< 0.001)	(<0.001)
Diabetes Tx (yes vs. no)	–	–	1.96±0.14	–	–	–	–	–
			(< 0.001)					
HTN Tx (yes vs. no)	–	–	–	13.3±1.80	–	–	–	–
				(< 0.001)	–			
Lipid Tx (yes vs. no)	–	–	–	–	–0.04±0.26	–0.58±0.44	0.06±0.19	–
					(0.873)	(0.182)	(0.742)	
Activity (×10^3^ Kcal/day)	0.88±0.11	1.86±0.26	–0.01±0.03	0.34±0.72	0.03±0.02	–0.04±0.03	–0.01±0.01	–0.05±0.03
	(< 0.001)	(< 0.001)	(0.677)	(0.635)	(0.132)	(0.255)	(0.313)	(0.129)
*R* ^2^	0.195	0.212	0.313	0.210	0.162	0.139	0.261	0.201
*n*	706	706	706	706	706	706	706	670

The statistics listed in each cell are the 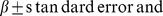 (*p* value) respectively.

The units and abbreviations are the same as those specified in Table 1. In addition, Tx  =  treatment and *R* ^2^  =  coefficient of determination.

* diabetics were excluded.

Diabetes Tx, HTN Tx and Lipid Tx denote previously receiving any medication respectively for treating diabetes, hypertension and lipid disorders.

**Table 3:** Multiple logistic regression analyses of the associations between vegetarian status and selected metabolic parameters as outcome variables using the subjective variable selection procedure based on pathophysiologic knowledge.

				Outcome	variable			
		Central						High
Covariate	Obesity	obesity	DM	Hypertension	Hyper TG	Hyper LDL-C	Hypo HDL-C	HOMA-IR*
Vegetarian (yes vs. no)	0.56 (0.38–0.81) (0.002)	0.39 (0.26–0.59) (< 0.001)	0.62 (0.30–1.27) (0.190)	0.91 (0.64–1.31) (0.618)	0.84 (0.55–1.29) (0.432)	0.23 (0.12–0.40) (< 0.001)	1.56 (1.08–2.28) (0.019)	0.71 (0.48–1.06) (0.094)
Nagelkerke *R* ^2^	0.202	0.165	0.203	0.219	0.129	0.129	0.274	0.138
HL GOF test *p* value	0.01	0.295	0.484	0.231	0.726	0.679	0.020	0.060
AUC	0.756	0.735	0.817	0.739	0.691	0.735	0.780	0.700
*n*	706	706	706	706	706	706	706	670

The statistics listed in each cell are the estimate of adjusted odds ratio, (95% confidence interval of adjusted odds ratio), and (*p* value of Wald test) respectively.

The units and abbreviations are the same as those specified in Table 1.

* The patients with diabetics were excluded.

The covariates on the variable list to be selected during variable selection procedure for control of confounding bias were those listed in Table 1. Specifically, the adjusted covariates in each multiple logistic regression model were the same as those in the corresponding multiple linear regression model (BMI, waist, glucose levels, systolic blood pressure, log(TG), LDL-C, HDL-C, and logHOMA-IR) in Table 2 respectively. For example, the adjusted covariates for obesity in Table 3 were the same as those for BMI in Table 2.

HL GOF test: Hosmer-Lemeshow goodness-of-fit test, where *p* value > 0.05 indicated a good fit of logistic regression model to data.

AUC: Area under receiver operating characteristic curve. AUC of 0.7 and above was considered acceptable discrimination.

Although the VEG group was associated with lower fasting plasma glucose than the non-VEG group in multi-variate linear regression (Table 2), it was not significantly associated with reduced risk for DM in the logistic regression analysis (Table 3) compared to the non-VEG. In both multi-variate linear and logistic regression models, the VEG was not associated with reduced SBP and reduced risk for hypertension respectively (Tables 2 & 3).

For lipid profiles, the VEG group was significantly associated with lower levels of LDL-C and HDL-C in multi-variate linear regression (Table 2) compared to the non-VEG. They also had reduced risk for high LDL-C and increased risk for low HDL-C in multi-variate logistic regression compared to the non-VEG shown in Table 3. On the other hand, the VEG group was associated with slight increase in TG with a borderline statistical significance compared to the non-VEG in multivariate linear regression (Table 2). It was not associated with reduced risk for high TG compared to the non-VEG in logistic regression (Table 3).

Further approach using the stepwise variable selection procedure to variable selection for linear regression analysis yielded the similar results like Table 2. Yet, the VEG group was even more strongly associated with higher plasma log(TG) than non-VEG group (data not shown). Approach using the stepwise variable selection procedure to variable selection for logistic regression analysis also yielded the similar results like Table 3 (data not shown). And, the area under receiver operating characteristic curves and the Hosmer-Lemeshow GOF tests were calculated for each logistic regression model.

Finally, the VEG group had 41% and 55% lower risk respectively for the MetS according to the modified NCEP definition and the IDF definition than the non-VEG group, after adjusting for age, menopause status, and activity score (data not shown). The vegetarian duration was further grouped into those ≥11 years and those >1 but <11 years (Table 4) for ovo-lacto-vegetarians. The VEG group with ovo-lacto-vegetarian duration ≥11 years and >1 but <11 years in general had reduced risk for the MetS using either definition after adjusting for age, menopause status, and activity score (Table 4) compared to the non-VEG. The pure vegetarians also had reduced risk for the MetS, but this did not reach statistical significance, probably because of its small sample size (*n*  =  22). The Hosmer-Lemeshow GOF tests were passed and AUCs above 0.7 were considered acceptable discrimination. The potential benefit does not appear to decline after 10 years. We also compared the characteristics of the MetS subjects between the VEG and non-VEG. The HDL-C was significantly lower in the VEG group with the MetS than the non-VEG group with the MetS (VEG: 1.11 mmol/L or 43.0 mg/dL; non-VEG: 1.21 mmol/L or 46.7 mg/dL, *p* = 0.024). The BMI was significantly lower in the VEG group than the non-VEG group (VEG: 25.2; non-VEG: 26.5, *p*  =  0.031) with the MetS. However, there was no significant difference in triglycerides, plasma glucose, waist circumference, and blood pressures between the VEG and non-VEG groups with the MetS.

**Table 4:** Multiple logistic regression analyses of the associations between polychotomous vegetarian status and selected metabolic parameters as outcome variables.

	Outcome	variable
Covariate	MNCEP MetS (*n* = 706)	IDF MetS (*n* = 706)
Ovo-lacto-vegetarian (1–11 years vs. Non-VEG)	0.55 (0.32–0.92)(0.026)	0.46 (0.26–0.79)(0.006)
Ovo-lacto-vegetarian (≥ 11 years vs. Non-VEG)	0.58 (0.33–0.10)(0.054)	0.43 (0.23–0.76)(0.005)
Pure vegetarian (yes vs. Non-VEG)	0.21 (0.01–1.09)(0.138)	0.22 (0.01–1.15)(0.152)
Age (years)	1.08 (1.05–1.12)(< 0.001)	1.08 (1.04–1.12)(< 0.001)
Menopause (yes vs. no)	0.96 (0.50–1.88)(0.902)	0.84 (0.43–1.67)(0.615)
Activity (× 10^3^ Kcal/day)	1.56 (1.28–1.92)(< 0.001)	1.84 (1.49–2.31)(< 0.001)
Nagelkerke *R* ^2^	0.139	0.170
HL GOF test *p* value	0.112	0.212
AUC	0.730	0.757

The statistics listed in each cell are the estimated adjusted odds ratio, (95% confidence interval of adjusted odds ratio), and (*p* value) respectively.

MNCEP MetS  =  modified national cholesterol education program criteria of the metabolic syndrome, IDF MetS  =  International Diabetes Federation criteria of the metabolic syndrome; non-VEG  =  non-vegetarian.

Vegetarian duration < 1 year was defined as “non-vegetarian” (Non-VEG) as the reference group. The *n* of non-vegetarians  =  315, *n* of ovo-lacto-vegetarians of 1–11 years  =  215, *n* of ovo-lacto-vegetarians ≥ 11 years  =  154, and *n* of pure vegetarian  =  22 respectively.

The vegetarian status includes 4 mutually exclusive categories, non-vegetarian, ovo-lacto-vegetarian for 1–11 years, ovo-lacto-vegetarian for ≥11 years, and pure vegetarian, which was defined by three dummy variables with non-vegetarian as the reference category.

HL GOF test: Hosmer-Lemeshow goodness-of-fit test, where *p* value > 0.05 indicated a good fit of logistic regression model to data.

AUC: Area under receiver operating characteristic curve. AUC of 0.7 and above is considered acceptable discrimination.

## Discussion

Consistent with previous studies, we found that the female vegetarians were associated with lower BMI, smaller waist circumference, lower levels of fasting plasma glucose, T-Chol, and LDL-C, but they also had lower HDL-C. So far, only one study in the literature specifically addressed the association of vegetarian diet with reduced risk for the MetS [12]. We found that indeed the female vegetarians, mainly lacto-ovo-vegetarians in our study, were associated with reduced risk for the MetS and IR. With further detailed analyses, we showed that the benefits can still be observed with a vegetarian duration over 10 years. Among the subjects with the MetS, the subjects in the VEG group appeared to have lower BMI, but also lower HDL-C compared to the non-VEG.

The lipid profile was also of great interest. The VEG group had lower total cholesterol, LDL-C, and HDL-C than the non-VEG group. Although lower HDL-C may appear unfavorable for the VEG in terms of vascular protection, the VEG group had lower T-Chol/HDL-C and LDL-C/HDL-C ratios than the non-VEG group as well. The ratio of T-Chol/HDL-C has been reported to be a better predictor for cardiovascular diseases than the total cholesterol or LDL cholesterol alone [20], [21], or even LDL-C/HDL-C ratio [22].

The TG/HDL-C ratio was previously suggested to be a surrogate predictor for IR [23]. In this study the VEG group had better insulin sensitivity without significant difference in TG/HDL-C ratio compared to the non-VEG group. The higher TG/HDL-C ratio in the VEG group in this study was mainly attributed to lower HDL-C. Reaven *et al.* also showed that the TG/HDL-C ratio did not correlate well with IR in the African Americans because this population might have relatively lower plasma triglyceride level [24]. These findings suggest that the TG/HDL-C ratio may not be a universal surrogate marker for IR. Special characteristics of the studied populations should be taken into account when using TG/HDL-C as a surrogate marker for insulin resistance.

Our results favoring that the vegetarian diet is associated with better metabolic profiles were consistent with many previous reports [25]. Because we did not record their dietary contents in this study, it is difficult for us to compare our results in details with the other dietary patterns. In fact, many previous reports about the beneficial effects of vegetarian diets, especially those from Western societies were from 7-day Adventist Christian Church [25]. Their vegetarians included several varieties (semi-, pesco-, lacto-ovo- and vegans). In contrast to this, most vegetarians in this study were lacto-ovo-vegetarian. In Buddhist teachings, semi- and pesco-vegetarians are not classified as vegetarians. In Asia, the majority of vegetarians practice this dietary habit for religious reasons. Therefore, this difference should be kept in mind when comparing vegetarian data between Western regions and Asia.

In the study by Rizzo *et al*.[12], the vegetarians were classified by the frequency of meat, poultry or fish consumption in a month or a week. In contrast, the vegetarian in our study did so for religious reason. The only animal products they consumed were milk and eggs for ovo-lacto-vegetarians. The dietary pattern is more restricted in our study than theirs. However, we did not record the frequency of milk or egg consumption in our study. Based on our understanding, they were usually consumed on daily basis for nutritional concern. Second, in our study we only analyzed women because much less men practiced vegetarian diet for religious reason in Taiwan. Third, although the conclusions were similar in both studies, the vegetarians in our study had lower mean level of HDL-C and higher mean level of triglyceride as compared with the non-vegetarians, whereas in Rizzo’s report their vegetarians had higher HDL-C and lower TG. Fourth, the prevalence of the MetS in our study was lower than that in their study. Last but not the least, we were able to report the effect of vegetarian duration on their risk of the MetS.

Not many vegetarian studies related to Buddhism were reported so far. Compared to our report, the sample sizes were often small, or the age or gender proportion was not matched in these studies. In a previous study from our hospital (19 vegetarians and 17 omnivores), the vegetarians were shown to have better insulin sensitivity [26]. A report from the other Buddhist hospital in Taiwan with 49 Buddhist lacto-vegetarian, the vegetarians had lower BMI and better insulin sensitivity compared to the omnivores [27]. Another study also from our hospital with 99 vegetarians reported lower total cholesterol, LDL-C and hs-CRP, but higher homocysteine in vegetarians [28]. An interesting study from South Korea comparing 54 Buddhist nuns and 31 omnivore Catholic nuns, the vegetarians seems to have higher BMI than the omnivores [29]. However, in vegetarians the body fat was inversely correlated with the duration of vegetarianism. In a study with 102 Taiwanese Buddhist nuns, vegetarians were found to have lower SBP and total cholesterol [30]. Because the vegetarian dietary patterns related to Buddhism may also be different among different religious subgroups and different geographical regions in Asia, further study on this issue is warranted.

This study has some limitations. First, we were unable to record detailed descriptions of daily dietary contents because of the heterogeneity in food preparation and serving in Taiwanese cuisine. Second, the subjects involved in this study were all female because there were few male vegetarians in our sample. Hence, the effects of vegetarian lifestyle might not be extrapolated to male gender. Third, we also analyzed the data of lacto- or ovo-vegetarians and obtained similar trends. However, they did not reach statistical significance, mainly because of small sample sizes.

## Conclusions

The lacto-ovo-vegetarian behavior related to Buddhism is associated with reduced risk for the MetS and IR and may potentially provide metabolic and cardiovascular protective effects in women. Further investigation of such benefits in both genders in prospective studies is warranted.
